# Differential neural responses to rhythmic and patterned TMS protocols: Insights from EEG spectral analysis

**DOI:** 10.1038/s41386-025-02306-w

**Published:** 2026-01-07

**Authors:** Thomas E. Valles, Mohamad Shamas, Hope Hawkins, Cole Matthews, Doan Ngo, Hrag Peltekian, Hewa Artin, Margaret G. Distler, Dustin Z. DeYoung, Evan H. Einstein, Nathaniel D. Ginder, Ralph J. Koek, David E. Krantz, Michael K. Leuchter, Hanadi A. Oughli, Aaron R. Slan, Thomas B. Strouse, Reza Tadayonnejad, Scott A. Wilke, Alexander S. Young, Juliana Corlier, Andrew F. Leuchter

**Affiliations:** 1 TMS Clinical and Research Service, Neuromodulation Division, Semel Institute for Neuroscience and Human Behavior at UCLA, Los Angeles, CA USA; 2 Department of Psychiatry and Biobehavioral Sciences, David Geffen School of Medicine at UCLA, Los Angeles, CA USA; 3 Division of Humanities and Social Sciences, California Institute of Technology, Pasadena, CA USA

**Keywords:** Translational research, Preclinical research

## Abstract

Repetitive Transcranial Magnetic Stimulation (rTMS) engages brain networks for the treatment of Major Depressive Disorder (MDD), using either rhythmic (e.g., 10 Hz) or patterned (e.g., intermittent Theta Burst, or iTBS) stimulation protocols. The distinct effects of these protocols on brain function are not well understood. Sixteen subjects with MDD underwent a TMS–electroencephalography (TMS–EEG) “interrogation” paradigm, in which a broad range of rhythmic and patterned stimulation frequencies were administered in a randomized order to the left dorsolateral prefrontal cortex (L-DLPFC). rTMS-induced changes in oscillatory activity and effective connectivity to the DLPFC were examined at each frequency. Linear mixed-effects models revealed widespread changes in power and connectivity, with magnitude and regional distribution of change dependent upon both protocol and frequency of stimulation. Increases in beta band power were most prominent with patterned stimulation, while rhythmic stimulation increased both alpha and beta power at stimulation frequencies greater than 7 Hz (*p* < 0.05). Source localization showed that patterned and rhythmic stimulation elicited activation in distinct subregions of the cingulate. Rhythmic and patterned stimulation also had distinct effects on connectivity: notably, only rhythmic stimulation increased connectivity with regions near the stimulation site, while only patterned stimulation decreased connectivity to the left precuneus. Both protocols increased the effective connectivity to the orbitofrontal cortex in the theta and beta response bands (*p* < 0.05). These results indicate that rhythmic and patterned rTMS engage distinct brain regions in a protocol- and frequency-dependent manner. Future studies should examine how these mechanistic differences may relate to clinical outcomes.

## Introduction

Repetitive Transcranial Magnetic Stimulation (rTMS) is an FDA-cleared intervention for treatment-resistant Major Depressive Disorder (MDD), with response rates up to 65% [1–3]. However, treatment outcomes vary substantially across patients, with many individuals deriving limited benefit [4, 5].

The two most common rTMS treatment protocols are rhythmic stimulation (RS; trains of pulses delivered at a uniform frequency, e.g., 10 Hz) and patterned stimulation (PS; 50 Hz triplet bursts modulated by a carrier frequency, e.g., 5 Hz or intermittent Theta Burst Stimulation [iTBS]) [6, 7]. While these protocols are equally efficacious for treatment of depression in populations of patients, evidence indicates that some patients will show clinical benefit from either rhythmic or patterned stimulation, but not both [8–10]. The lack of response to rTMS treatment in some individuals is believed to reflect the failure of stimulation to engage brain networks [11], which appears to be dependent on the stimulation parameters including protocol, target, and frequency of stimulation [12].

In healthy subjects, the extent to which particular networks are engaged depends in part upon the specific stimulation frequency [13]. In patients with MDD, different rhythmic frequencies have been shown to elicit distinct changes in connectivity, with the degree of change in connectivity originating at the stimulation site being positively associated with treatment outcomes [14]. These findings are consistent with preclinical work in primates, in which specific frequencies engage select neural networks [15], as well as in rodents, where patterned and rhythmic stimulation differentially affect the pattern of immediate early gene expression in the brain [16].

Neural oscillations have been attributed a critical role in the processing of behavioral and emotional states [17] and in communication among neuronal assemblies through the synchronization of presynaptic activation pattern [18]. Oscillations also have been found to be dysregulated in pathological conditions [19, 20]. For example, patients with MDD exhibit altered oscillatory profiles particularly in theta and alpha, but also beta bands [21, 22]. Because rTMS has been shown to alter both oscillatory power and connectivity in a frequency dependent manner [13, 23–25], it may offer a method for modifying abnormal network activity patterns and ameliorate mood symptoms [26]. We therefore conducted this study to identify how different combinations of stimulation frequency and protocol differentially affect neurophysiologic function.

While some earlier work has demonstrated that patterned and rhythmic stimulation induce differential changes in cortico-striatal connectivity in MDD [27], no study has broadly and systematically examined the effect of stimulation protocol (and frequency within a protocol) on engagement of brain networks or regions. To address this question, we performed a within-subject examination of differences in EEG oscillatory power and effective connectivity in response to 97 different rhythmic and patterned trains of stimulation. Our goal was to examine whether there were distinct effects of rhythmic and patterned protocols, as well as of different frequencies within a protocol, on activation of brain areas and alteration of brain connectivity.

## Materials and methods

### Patient demographics and TMS protocol

Subjects were 16 patients (mean age ± standard deviation: 46.5 ± 18.3, 8 M and 8 F) who were treated at the UCLA TMS Clinical and Research Service, who met DSM-5 criteria for MDD confirmed by the MINI [28], and had failed to benefit from at least three trials of antidepressant medication therapy. Subjects had an average baseline Hamilton Depression Rating Scale (HDRS) score of 18.7 ± 5.2. At the time of the TMS session, or within the preceding three months, 11 patients were using medications that could affect neural activity. These medications were grouped into five categories: anxiolytics, antidepressants, mood stabilizers, psycho-stimulants, and hormones (see Table S1 for subject counts for each category). Exact permutation tests revealed that there were no significant differences in the motor thresholds of patients who were using medication versus those who were not in all categories except mood stabilizers (*p* = 0.005). We compensated for any potential disparities in neuronal excitability associated with medication use by standardizing stimulation intensity to 100% of the motor threshold. See Supplementary Materials and Methods Section 1 for further details. All study procedures were conducted in accordance with UCLA Institutional Review Board approval and relevant ethical guidelines. Participants provided informed consent before any study-related procedures were initiated.

rTMS was administered using procedures previously reported in detail [14] and described briefly here. The coil was placed over the left DLPFC using the Beam-F3 method [29] and TMS was delivered using the Magstim Super Rapid Plus 1 with a 70 mm Double Air Film coil (Magstim, Whitland South Wales, UK) for all subjects. Motor thresholds (MTs) were determined by identifying the minimum stimulation intensity required to elicit a motor evoked potential (EMG) in the right abductor pollicis brevis (APB) or first dorsal interosseus (FDI) in at least 50% of applied stimuli. Since this was the first rTMS session for each participant, stimulation was administered at 100% of their motor threshold to ensure tolerability. During the session, participants were seated at a semi-reclined position, employing standard safety protocols including ear protection.

**Fig. 1:**
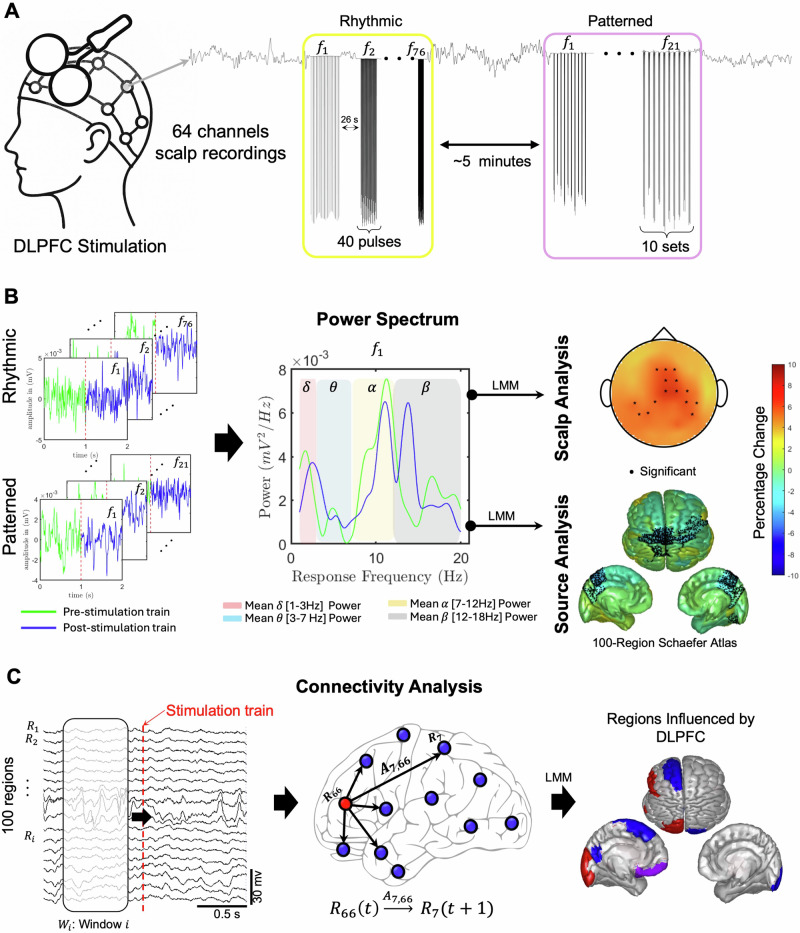
Data Collection and Processing Pipeline. **A** Data were collected from 16 MDD subjects using a 64-channel TMS-compatible EEG headcap during a single stimulation session. The protocol consists of rhythmic stimulation trains at random frequencies, followed by an approximate five-minute rest period, after which trains of patterned stimulation are administered. Rhythmic stimulation trains consisted of 40 pulses, with frequencies between 3 and 18 Hz with 0.2 Hz steps administered in a randomized order. Patterned stimulation trains consisted of 10 sets of 50 Hz triplets, delivered at a frequency between 3 and 7 Hz in 0.2 Hz intervals administered in a randomized order. **B** Recorded EEG was preprocessed to remove artefacts and identify epochs pre and post each train of stimulation for both rhythmic and patterned paradigms. Percentage change in the power spectrum was calculated and significant changes in response frequency bands were determined from both scalp and source signals using linear mixed models. **C** The Influence of the DLPFC on all regions was calculated using dynamic network models. Regions that showed a significant change before and after stimulation were determined using a linear mixed model and plotted on source space.

The interrogation protocol [14] consisted of rhythmic and patterned segments (Fig. 1A). In the rhythmic segments, patients received 76 40-pulse trains of TMS with an inter-train-interval (ITI) of 26 s. Rhythmic stimulation frequencies between 3-18 Hz in 0.2 Hz increments were administered in a randomized order, with the range of frequencies chosen to encompass commonly used high-frequency rTMS protocols [30]. After a break of approximately five minutes, the patterned segment of the interrogation was administered with 21 trains of patterned TMS with an ITI of 26 s. Each train consisted of 10 sets of 50 Hz triplets, delivered at a frequency between 3 and 7 Hz in 0.2 Hz intervals in a randomized order. This range was centered on the standard 5 Hz frequency used for iTBS with frequencies within ±2 Hz added to coincide with conventional definitions of the theta frequency band. For both paradigms, the 0.2 Hz step size was selected to capture potentially distinct frequency-dependent response patterns at a fine scale.

### EEG recording

64-channel electroencephalographic (EEG) data were collected during the interrogation using the “eego mylab” system (Advanced Neuro Technology {ANT}; Enshede, Netherlands) at a sampling rate of 2000 Hz. Electrodes were placed using the 10-20 system with the ground electrode positioned at AFz and the reference electrode at CPz. Impedances were kept below 20 kΩ.

### EEG pre-processing and power calculation

1 second pre- and 2 s post-TMS trains were concatenated to create 3-s long epochs, though only data up to one second post-train was used for analysis. Data immediately before and after the pulses (-5 ms : 20 ms) were replaced with Gaussian noise to mimic brain signal. Remaining TMS pulse artifacts were removed by detrending the signal using a Piecewise Cubic Hermite Interpolating Polynomial (PCHIP).

The FASTER pipeline [31] was used to automatically remove eye, muscle, heart and other artifacts. The pipeline included bandpass filtering (1–55 Hz), resampling to 1 kHz, re-referencing to common average montage, ICA-based artifact removal, and channel rejection. Data were then visually inspected and epochs which still had visible non-physiological noise or artifacts were rejected (see Supplementary Materials and Methods Section 2 for more details regarding the EEG preprocessing pipeline).

The Gabor transform was used to compute spectral power with a window width of 3 cycles per frequency (see Supplementary Materials and Methods, Section 3). The frequency spectrum for pre- and post-stimulation was obtained by averaging the spectrogram across the one-second windows pre- and post-stimulation respectively. Relative power was computed by normalizing the spectra values to their total sum.

Response frequencies (i.e., the frequencies at which power and connectivity were measured using the Gabor transform) were categorized into four distinct bands: delta (1–3 Hz), theta (3–7 Hz), alpha (7–12 Hz), and beta (12–18 Hz).

### Source localization

To increase the estimation accuracy of the spatial properties of scalp EEG signals, individual EEG electrode positions were digitized (Brainsight; Rogue Research Inc., Montreal, Canada) and co-registered to a standard head model (Brainstorm; Tadel et al., 2011). The inverse solution was calculated using the weighted minimum norm estimate (wMNE) with fixed dipole orientation and standardized low-resolution brain electromagnetic tomography (sLORETA) to compute deep structures contributions by adjusting the noise covariance matrix using a regularization parameter. The inverse problem in EEG source localization is fundamentally ill-posed because multiple different configurations of neural sources within the brain can generate identical electrical potential patterns observed on the scalp [32]. This ambiguity means that, without additional constraints or assumptions, such as hypotheses about specific spatial or temporal characteristics of the signal origin, there is no unique solution for determining the exact brain sources responsible for the measured scalp activity. The noise covariance matrix was computed from data that was recorded during a resting state baseline period.

After solving the inverse problem, reconstructed sources were represented in a matrix of size $M\times {T}_{{samples}}$, where $M={\mathrm{15,002}}$ is the number of dipoles and ${T}_{{samples}}$ is the total number of samples. Dipoles were then grouped into 100 brain regions using a Schaefer-100 Atlas [33] (i.e., the signal space was reduced from $M\times {T}_{{samples}}$ to $100\times {T}_{{samples}}$ using principal component analysis) to construct the signals at the centroid of each of the 100 regions. See Supplementary Materials and Methods, Section 4 for more details regarding source localization.

### Effective connectivity

To assess the influence of the DLPFC (Atlas region #66) on all other 99 regions in the Schaefer atlas, the directional effective connectivity from the DLPFC to every other brain region was computed using a dynamical network model (DNM) [34]. In brief, Dynamical Network Models (DNMs) used generative models to characterize how each EEG source dynamically influenced the rest of the EEG network. The DNM was formulated as a linear time-varying (LTV) model, mathematically describing interactions between observed brain regions (EEG signals).

The absolute values of effective connectivity were computed for pre- and post-stimulation periods, separately for all response bands and subsequently normalized between 0 and 1 to ensure comparability across subjects and conditions. See Supplementary Materials and Methods, Section 5 for more details regarding the calculation of effective connectivity.

### Statistical analysis

Linear mixed models (LMMs) were employed to assess whether EEG sensor-level power, source-level power, and effective connectivity in specific response bands (delta, theta, alpha, and beta) differed significantly between pre- and post-stimulation conditions. Rhythmic stimulation frequencies were grouped into three ranges: theta-rhythmic stimulation (theta-RS; 3–7 Hz), alpha-rhythmic stimulation (alpha-RS; 7–12 Hz), and beta-rhythmic stimulation (beta-RS; 12–18 Hz). All patterned stimulation frequencies (3–7 Hz) were considered as one group (PS).

Models were run separately on each channel and source for every stimulation frequency range at each response frequency band. Each model had the form:

$${Value} \sim 1+{Timepoint}+{Stimulation\; Frequency}+\left(1|{Patient\; ID}\right)$$

where “${Value}$” denotes the power or effective connectivity measurement, “${Timepoint}$” designates a binary variable indicating whether the measurement came before or after stimulation, and “${Stimulation \ Frequency}$” indicates the stimulation frequency, rounded to the nearest integer and encoded as a categorical variable. Patient ID was encoded as a random effect to account for inter-subject variability.

The p-value corresponding to “${Timepoint}$” variable was used to determine whether the stimulation frequency had a significant effect on power or connectivity. The coefficient estimate for timepoint variable was used to determine whether the stimulation induced an increase or decreased in the output power or connectivity measure. P-values were corrected over channels/sources for multiple comparisons using the Benjamini-Hochberg procedure with a false discovery rate of 0.05 [35].

## Results

### Sensor-level power differences

**Fig. 2:**
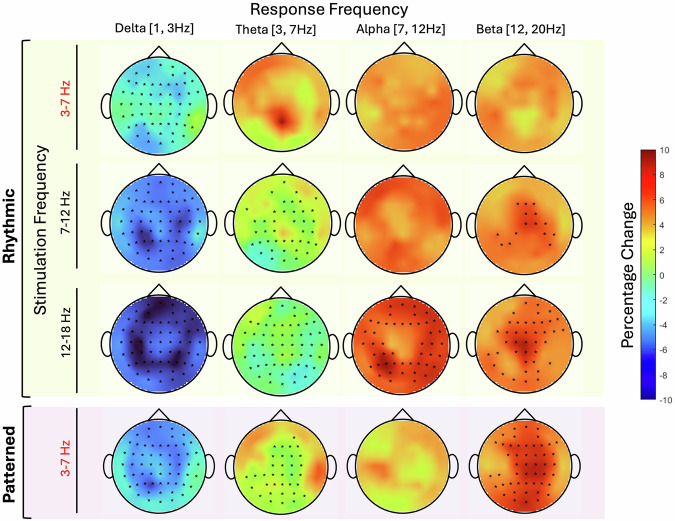
Scalp topographical mapping of percentage change in EEG power following rhythmic and patterned stimulation paradigms across frequency bands. The difference in power pre-post stimulation was plotted on the scalp axial projection, with each row corresponding to a specific stimulation frequency band. The first three rows represent rhythmic stimulation at theta-RS (3–7 Hz), alpha-RS (7–12 Hz) and beta-RS (12–20 Hz) while the fourth row illustrates PS. Columns represent the response frequency bands (from left to right): delta (1–3 Hz), theta (3–7 Hz), alpha (7–12 Hz) and beta (12–20 Hz). Scalp regions are color-coded indicating percentage changes in power ranging from -10% (blue) to +10% (red). Regions exhibiting statistically significant pre-post differences, as determined by the linear mixed model, are marked with black stars.

RS and PS exhibited several shared effects on response band power across stimulation frequencies. There were significant and widespread decreases in delta response band power following all stimulation protocols, including PS (64 channels), theta-RS (61 channels), alpha RS (64 channels), and beta-RS (64 channels). Beta-RS induced the strongest decrease with a nearly 10% average delta response band power reduction in the prefrontal cortex (PFC) (Fig. 2, Table S2).

Widespread decreases in the theta response band also were seen after most protocols, including alpha-RS (33 channels), beta-RS (59 channels) and PS (18 channels). The percent change in theta response band power ranged between -4.6% and -0.02%. In contrast, theta-RS did not elicit significant changes in theta response band power.

Changes in some response bands were distinct to particular protocols and stimulation frequencies. Alpha response band power increases were only observed following beta-RS (50 channels), with no significant changes in this response band observed after any of the other protocols. In contrast, the most widespread increases in beta response band power were induced by PS (51 channels), affecting most regions except the left fronto-central region (FT7, T7, FC5, C5, C3, CP3), and right temporal and temporo-parietal regions (F8, FT8, T8, TP8, and P8), followed by beta-RS (41 channels) and alpha-RS (15 channels). Theta-RS did not induce any significant changes in beta response band power.

### Source power differences

**Fig. 3:**
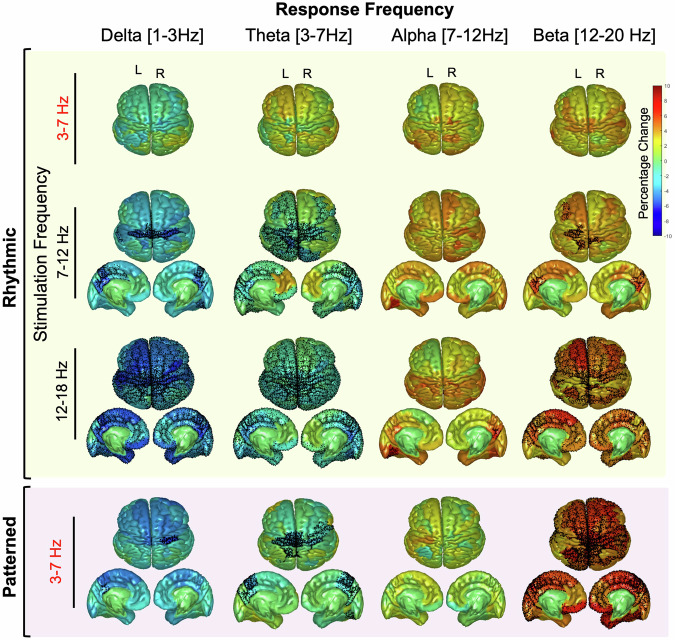
Source topographical mapping of percentage change in EEG power following rhythmic and patterned stimulation paradigms across frequency bands. The difference in power pre-post stimulation was plotted in source space on a cortical parcellation based on Schaefer 100 atlas. Each row corresponds to a specific stimulation frequency band. The first three rows represent rhythmic stimulation at theta-RS (3–7 Hz), alpha-RS (7–12 Hz) and beta-RS (12–20 Hz) while the fourth row illustrates patterned stimulation (PS). Columns represent the response frequency bands (from left to right): delta (1–3 Hz), theta (3–7 Hz), alpha (7–12 Hz) and beta (12–20 Hz). Each subplot includes an axial top view of the brain, with color-coded regions indicating percentage changes in power ranging from -10% (blue) to +10% (red), accompanied by sagittal views of the inner left and right hemispheres. Regions exhibiting statistically significant pre-post differences, as determined by the linear mixed model, are marked with black dots.

Source localized power changes elicited within particular response bands were largely consistent with the changes seen in sensor-based analyses. A widespread significant decrease in delta response band power (*p* < 0.05 after FDR correction) was induced by all protocols except for theta-RS. Source-based analyses revealed significant regional variation, however, with alpha-RS inducing decreases in delta response band power in 19, primarily precentral, regions, including somatomotor areas, the precuneus, bilateral caudal and dorsal cingulate, and left rostral and perigenual cingulate (Fig. 3, Table S3). Beta-RS induced widespread decreases in delta response band power in similar regions but on a wider scale (95 of the 100 regions). PS showed decreases in 3 regions, namely the left posterior cingulate and 2 other precentral regions.

Theta response band power was affected similarly to delta response band power, but on a much wider scale. In fact, alpha-RS elicited decreases theta response band power in 63 regions, including most regions of bilateral cingulate, as well as the precuneus, parietal, prefrontal and occipital right and left lobes. Similarly, beta-RS induced a widespread decrease in the theta response band power (95 regions). PS induced decreases in 23 regions (bilateral posterior cingulate), bilateral caudal anterior cingulate, prefrontal cortex, left and right precuneus, as well as some somatomotor areas.

The alpha response band was not affected by any stimulation protocol except for beta-RS in two regions. Alpha response band power increased by 6–7% in the right precuneus and the left occipital lobe.

Beta response band power increased following all stimulation protocols except theta-RS. Alpha-RS stimulation induced increased beta response band power in 11 regions, including right and left caudal cingulate, precuneus, and precentral regions. Both beta-RS and PS induced a widespread increase in beta response band power (59 and 64 regions respectively).

### Effective connectivity differences

**Fig. 4:**
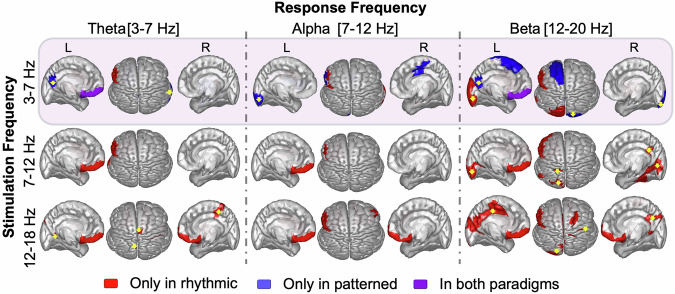
Pre-Post Changes in DLPFC influence on cortical regions following rhythmic and patterned stimulation paradigms. The Schaefer-100 atlas regions are color coded: red indicates significant changes exclusively under rhythmic stimulation, blue under patterned stimulation, violet under both paradigms, and white showed no significant change. Rows represent (from top to bottom) the stimulation frequencies theta (3–7 Hz), alpha (7–12 Hz) and beta (12–18 Hz), and columns represent (from left to right) the response frequencies theta, alpha and beta. Each subplot displays a top axial view and parasagittal views of left and right hemispheres. Changes in the negative direction (i.e. the influence of DLPFC on the highlighted regions is significantly less after stimulation) are indicated by a yellow arrow overlaid on the region; changes otherwise represent positive changes.

Both patterned and rhythmic stimulation protocols (in particular theta-RS and beta-RS) elicited significant increases in the effective connectivity of left DLPFC to the orbitofrontal cortex (OFC) and subgenual anterior cingulate (sgACC) (Fig. 4, Table S4). This enhancement was observed in both theta and beta response bands, indicating a robust modulation of DLPFC–OFC and DLPFC-sgACC connectivity across multiple oscillatory domains.

PS was uniquely associated with significant decreases in connectivity to the left precuneus (theta and beta response band), right frontal operculum (theta response band), and the left and right occipital lobes (alpha and beta response bands). An increase in connectivity was seen locally within the prefrontal cortex (beta response band), to the left rostral ACC, left insula, and left occipital lobe (alpha response band).

RS was associated with many more unique connectivity changes than PS, with differential effects by stimulation frequency. Across the brain, increasing the frequency of RS led to more widespread connectivity changes. For example, across the theta, alpha, and beta response bands, theta-RS elicited significant changes in 23 regions, while beta-RS affected 43 regions. Additionally, connectivity in higher response frequencies was more often modulated than that in lower response frequencies. Across all stimulation protocols, significant changes in the theta response band were observed in 26 regions, while significant changes in the beta response band were observed in 40 regions.

Theta- and alpha-RS modulated connectivity of the left PFC to the stimulation site across all response frequency bands by an average of 20.7%. Common effects across all response bands also were seen with theta-, alpha-, and beta-RS all increasing connectivity to left OFC and sgACC by an average of 20.9% (except for theta-RS in the alpha response band).

A number of distinct regional effects dependent upon the specific stimulation frequency band were also observed. Alpha-RS uniquely increased connectivity to the left dorsal precentral gyrus (27.3%) in the theta response band and reduced connectivity to bilateral posterior and occipital cortices in the beta response band. In the alpha response band, theta-RS uniquely increased connectivity to right frontal operculum and beta-RS elicited the greatest number of unique network changes including with right ventrolateral precentral gyrus, bilateral insula, and two occipital lobe regions. Beta-RS also resulted in extensive increased connectivity with the posterior cingulate and parietal regions in the beta response band. Overall, as stimulation frequency increased from theta to beta, the changes in connectivity became more widespread, especially in high frequency response bands.

## Discussion

These findings demonstrate that patterned and rhythmic rTMS administered to the left DLPFC in patients with MDD produce widespread effects on brain activity and connectivity. There were significant differences in regional patterns of activation, as well as increases and decreases in connectivity, depending on the specific stimulation protocol and stimulation frequency applied. PS and RS elicited some similar patterns of change in regional activation (e.g., decreases in delta and theta power, increases in beta power). However, stimulation with each protocol was most commonly followed by distinct, differential regional changes (e.g., PS most prominently affecting connectivity to the precuneus and subregions of the cingulate and RS most commonly affecting connectivity within the PFC). Parallel findings were seen with the effect of protocol and frequency on connectivity, with some effects common to both protocols (e.g., increased OFC connectivity with both RS and PS). RS in general elicited greater connectivity changes than PS, particularly in the beta stimulation band, but PS elicited distinct regional changes affecting specific regions that have been implicated in the pathophysiology of MDD such as the prefrontal cortex, left rostral ACC, and left insula.

Within each protocol, the effects of stimulation differed topographically according to the frequency of stimulation. Most notably, faster RS frequencies (e.g., beta-RS) elicited the most widespread changes. Together, these findings highlight the relevance of both protocol and frequency in engaging brain networks and suggest that tailoring stimulation parameters may represent an opportunity to optimize rTMS effects.

The results of our investigation demonstrated that the effects of stimulation were widespread, affecting multiple distant cortical and subcortical brain areas in the first second after stimulation offset. Peak effects were often located at sites distant from and even contralateral to the left DLPFC target. It is important to note that the distribution of stimulation effects on connectivity was not homologous with any established resting-state or functional networks. Instead, the most prominent connectivity changes affected specific regions including the orbitofrontal cortex, precuneus, dorsal ACC, and sgACC, which can be considered components of the default mode, salience, cognitive control, and other networks [36].

While several prior studies have examined the effects of repetitive stimulation, none have systematically contrasted both protocol and frequency effects. Consistent with previous reports, the present study demonstrated that PS affected theta–alpha amplitude ranges, while RS appeared to selectively modulate alpha carrier frequencies [37]. The current study extends these findings, as we observed that only beta-RS produced measurable changes in alpha and beta power. This suggests that to elicit a response in a certain frequency, the stimulation frequency may need to be the same or faster than the desired response frequency. This difference from prior work may reflect the fact that the present study directly contrasted pre- and post-stimulation brain states in a frequency-dependent manner within the same session.

We also observed that response frequencies were not usually modulated by their corresponding stimulation frequency in a direct input/output relationship. Though stimulation frequencies did sometimes induce changes in the parallel response band (e.g., beta-RS increasing beta response band power), changes were often also seen in other response bands (e.g., theta-RS increasing beta response band power). Notably, theta-RS and PS did not always elicit the same response pattern, despite sharing the same underlying theta frequency. PS produced distinct and robust pre-post differences mainly in the theta and beta response bands, suggesting that patterned stimulation was more similar to fast rhythmic stimulation than slow rhythmic stimulation. At higher stimulation frequencies, rhythmic protocols induced modulation in deeper and more integrative regions such as the cingulate cortex, precuneus, and limbic areas, in agreement with previous findings [38–40]. While patterned stimulation also affected these networks, the effects were more spatially restricted. Given that increased EEG power at a specific frequency is generally interpreted as reflecting stronger synchronization of local neuronal populations [41], our results suggest that rhythmic and patterned stimulation protocols engage partially distinct neural networks, with the nature and extent of this engagement being tightly coupled to stimulation frequency.

These findings indicate that it is possible to modulate activity in distinct and divergent brain regions through a single stimulation site (in this case the left DLPFC) by modifying the protocol and frequency of stimulation. Prior work has shown increases in middle beta-band connectivity between the left DLPFC and fronto-limbic regions as well as reductions in gamma-band connectivity with anterior cingulate cortex [40]. While both 10 Hz rTMS and iTBS targeting the left DLPFC have been shown to enhance cortico-striatal connectivity [14], we were unable to make conclusive statements about the cortico-striatal connectivity in our study due to the ill-posed nature of the source localization inverse problem and the increasing localization error with depth [42, 43]. Extending this principle beyond the prefrontal cortex, 10 Hz rTMS and iTBS applied over the motor cortex in Parkinson’s disease patients have been demonstrated to differentially modulate large-scale network topology, including sensorimotor and default mode network connectivity [44], supporting the notion that stimulation parameters shape distributed network dynamics. In our study, we extend these findings by showing that the functional influence of DLPFC on distributed cortical areas, including the cingulate cortex, precuneus, frontal poles, and occipital lobe, is highly dependent on the frequency-specific stimulation paradigm employed. Notably, both rhythmic and patterned stimulation increased DLPFC-driven connectivity to the OFC, a region tightly linked to affective valuation and reward processing, in the theta and beta response bands. These results are consistent with growing evidence that beta oscillations play a key role in mediating prefrontal-limbic interactions during neuromodulation [40].

The approach of accessing multiple networks through a single common neuroanatomic target stands in contrast to the functional imaging approach in which fMRI is used to select distinct, individualized DLPFC sites that have specific patterns of connectivity with other regions (e.g., anticorrelated connectivity with sgACC) [11, 45]. Alternative stimulation targets also have been proposed based upon a combination of more pronounced anxiosomatic or dysphoric features and functional connectivity [46]. Future studies should further investigate whether a frequency and protocol-based targeting approach may be viable for targeting specific circuit dysfunctions or symptoms and enhancing treatment efficacy.

A growing literature suggests that rTMS-induced changes in connectivity may serve as biomarkers for clinical outcome [14, 47, 48]. Given the variable responses to different frequencies and protocols across regions shown here, future studies could seek to leverage an interrogation-like paradigm to identify a set of frequency/protocol combinations that most effectively elicit desired changes in specific EEG metrics within targeted brain regions. This approach could be applied not only to MDD, but also to other disorders with distinct neuropathophysiologic profiles. At a more individualized level, the interrogation protocol could be used to determine, for each patient, the frequencies and stimulation protocols that maximally modulate neural activity within relevant brain networks. Such a symptom- or disease-specific mapping approach could enable precision rTMS, tailoring treatment to the unique neurophysiologic or symptomatic profile of each patient. By linking frequency- and protocol- specific neural responses to behavioral or clinical outcomes, this strategy could enable evidence-based selection of stimulation parameters based on established biomarkers, while simultaneously facilitating the discovery of novel biomarkers of rTMS responsiveness.

Despite the strengths of our study, several limitations should be acknowledged. First, we examined changes in connectivity pre-post individual pulse trains of rTMS. It is not clear how changes in connectivity after a pulse train relate to changes that might be seen following a full treatment session, or a full course of rTMS treatment. Second, our stimulation paradigm grouped conditions based on stimulation frequency. While this grouping approach allowed for an input-output matching of stimulation and response frequencies, it could have obscured differences in responses between frequencies that are numerically close but functionally distinct. This design decision reflected the challenge of balancing the goal of sampling a wide range of stimulation parameters with collecting sufficient data at each condition to generate reliable estimates. While administering more trains at each individual frequency might have allowed us to elicit further response differences, it would have been significantly more burdensome for the subjects. Conversely, limiting the number of frequencies tested but increasing the number of trains at each frequency allows for more robust estimation within each group, but risks missing potentially important distinctions between closely spaced frequencies. Third, although we examined both patterned and rhythmic stimulation within the same session to enable within-subject comparisons, this design may have introduced carry-over effects or interactions among frequencies or protocols. The fact that stimulation frequencies were administered in random order to each individual subject, however, served to average out carry-over effects across subjects. Fourth, this study was conducted in a limited number of subjects with depression (*N* = 16) who were simultaneously receiving other antidepressant treatments. It is possible that different effects might be seen in MDD patients receiving other (or no) treatments, or in healthy control subjects. Finally, although our findings offer mechanistic insights into the effects of frequency and protocol on connectivity, the study did not examine the relationship of connectivity change to clinical outcomes. Future studies should incorporate longitudinal follow-up, and multimodal neuroimaging to clarify the causal and clinical significance of these network-level modulations.

## Conclusion

Our findings demonstrate that patterned and rhythmic stimulation administered to left DLPFC engaged neural circuits and modulated brain oscillations in distinct ways, dependent both upon the protocol and the frequency of stimulation administered. The elicited changes in activation and connectivity affected numerous brain areas that have been reported to be involved in the pathophysiology of MDD. These findings lay the groundwork for future research examining the relationship between protocol- and frequency-specific activation and connectivity profiles and rTMS clinical outcome.

## Supplementary information

Supplementary Materials and Methods

Table S2

Table S3

Table S4

## Data Availability

The data that support the findings of this study are available from the corresponding author, upon reasonable request.

## References

[1] Carpenter LL, Janicak PG, Aaronson ST, Boyadjis T, Brock DG, Cook IA, et al. Transcranial magnetic stimulation (TMS) for major depression: A multisite, naturalistic, observational study of acute treatment outcomes in clinical practice. Depress Anxiety. 2012;29:587–96. 22689344 10.1002/da.21969

[2] van Rooij SJH, Arulpragasam AR, McDonald WM, Philip NS. Accelerated TMS - moving quickly into the future of depression treatment. Neuropsychopharmacology. 2024;49:128–37. 37217771 10.1038/s41386-023-01599-z PMC10700378

[3] Cappon D, den Boer T, Jordan C, Yu W, Metzger E, Pascual-Leone A. Transcranial magnetic stimulation (TMS) for geriatric depression. Ageing Res Rev. 2022;74:101531. 10.1016/j.arr.2021.101531 PMC8996329 34839043

[4] Fitzgerald PB, Hoy KE, Anderson RJ, Daskalakis ZJ. A STUDY OF THE PATTERN OF RESPONSE TO rTMS TREATMENT IN DEPRESSION. Depress Anxiety. 2016;33:746–53. 27059158 10.1002/da.22503

[5] Klooster DCW, Ferguson MA, Boon PAJM, Baeken C. Personalizing Repetitive Transcranial Magnetic Stimulation Parameters for Depression Treatment Using Multimodal Neuroimaging. Biol Psychiatry Cogn Neurosci Neuroimaging. 2022;7:536–45. 34800726 10.1016/j.bpsc.2021.11.004

[6] Blumberger DM, Vila-Rodriguez F, Thorpe KE, Feffer K, Noda Y, Giacobbe P, et al. Effectiveness of theta burst versus high-frequency repetitive transcranial magnetic stimulation in patients with depression (THREE-D): a randomised non-inferiority trial. Lancet. 2018;391:1683–92. 29726344 10.1016/S0140-6736(18)30295-2

[7] Lan XJ, Yang XH, Qin ZJ, Cai D Bin, Liu QM, Mai JX, et al. Efficacy and safety of intermittent theta burst stimulation versus high-frequency repetitive transcranial magnetic stimulation for patients with treatment-resistant depression: a systematic review. Front Psychiatry. 2023;14:1244289. 10.3389/fpsyt.2023.1244289 PMC10423820 37583841

[8] Chen L, Thomas EHX, Kaewpijit P, Miljevic A, Hahn L, Lavale A, et al. Does switching between high frequency rTMS and theta burst stimulation improve depression outcomes?. Brain Stimul. 2022;15:889–91. 35714945 10.1016/j.brs.2022.06.005

[9] Citrenbaum C, Corlier J, Ngo D, Vince-Cruz N, Wilson A, Wilke SA, et al. Pretreatment pupillary reactivity is associated with differential early response to 10 Hz and intermittent theta-burst repetitive transcranial magnetic stimulation (rTMS) treatment of major depressive disorder (MDD). Brain Stimul. 2023;16:1566–71. 37863389 10.1016/j.brs.2023.10.006

[10] Slan AR, Citrenbaum C, Corlier J, Ngo D, Vince-Cruz N, Jackson NJ, et al. The role of sex and age in the differential efficacy of 10 Hz and intermittent theta-burst (iTBS) repetitive transcranial magnetic stimulation (rTMS) treatment of major depressive disorder (MDD). J Affect Disord. 2024;366:106–12. 39187197 10.1016/j.jad.2024.08.129

[11] Fox MD, Buckner RL, White MP, Greicius MD, Pascual-Leone A. Efficacy of transcranial magnetic stimulation targets for depression is related to intrinsic functional connectivity with the subgenual cingulate. Biol Psychiatry. 2012;72:595–603. 22658708 10.1016/j.biopsych.2012.04.028 PMC4120275

[12] Caulfield KA, Brown JC. The Problem and Potential of TMS’ Infinite parameter space: a targeted review and road map forward. Front Psychiatry. 2022;13:867091. 10.3389/fpsyt.2022.867091 PMC9127062 35619619

[13] Okazaki YO, Nakagawa Y, Mizuno Y, Hanakawa T, Kitajo K. Frequency- and Area-Specific Phase Entrainment of Intrinsic Cortical Oscillations by Repetitive Transcranial Magnetic Stimulation. Front Hum Neurosci. 2021;15:608947. 10.3389/fnhum.2021.608947 PMC7994763 33776666

[14] Leuchter AF, Wilson AC, Vince-Cruz N, Corlier J. Novel method for identification of individualized resonant frequencies for treatment of Major Depressive Disorder (MDD) using repetitive Transcranial Magnetic Stimulation (rTMS): A proof-of-concept study. Brain Stimul. 2021;14:1373–83. 34425244 10.1016/j.brs.2021.08.011

[15] Salinas FS, Franklin C, Narayana S, Szabó CÁ, Fox PT. Repetitive Transcranial Magnetic Stimulation Educes Frequency-Specific Causal Relationships in the Motor Network. Brain Stimul. 2016;9:406–14. 26964725 10.1016/j.brs.2016.02.006 PMC5385705

[16] Aydin-Abidin S, Trippe J, Funke K, Eysel UT, Benali A. High- and low-frequency repetitive transcranial magnetic stimulation differentially activates c-Fos and zif268 protein expression in the rat brain. Exp Brain Res. 2008;188:249–61. 18385988 10.1007/s00221-008-1356-2

[17] Bastos AM, Vezoli J, Fries P. Communication through coherence with inter-areal delays. Curr Opin Neurobiol. 2015;31:173–80. 25460074 10.1016/j.conb.2014.11.001

[18] Fries P. Rhythms for Cognition: Communication through Coherence. Neuron. 2015;88:220–35. 26447583 10.1016/j.neuron.2015.09.034 PMC4605134

[19] Fingelkurts AA, Fingelkurts AA. Altered structure of dynamic electroencephalogram oscillatory pattern in major depression. Biol Psychiatry. 2015;77:1050–60. 25662102 10.1016/j.biopsych.2014.12.011

[20] Uhlhaas PJ, Singer W. Neuronal Dynamics and Neuropsychiatric Disorders: Toward a Translational Paradigm for Dysfunctional Large-Scale Networks. Neuron. 2012;75:963–80. 22998866 10.1016/j.neuron.2012.09.004

[21] Fuggetta G, Pavone EF, Fiaschi A, Manganotti P. Acute modulation of cortical oscillatory activities during short trains of high-frequency repetitive transcranial magnetic stimulation of the human motor cortex: A combined EEG and TMS study. Hum Brain Mapp. 2008;29:1–13. 17318833 10.1002/hbm.20371 PMC6870897

[22] Özçoban MA, Tan O. Electroencephalographic markers in Major Depressive Disorder: insights from absolute, relative power, and asymmetry analyses. Front Psychiatry. 2024;15:1480228. 10.3389/fpsyt.2024.1480228 PMC11770048 39872429

[23] Mitoma R, Tamura S, Tateishi H, Mitsudo T, Tanabe I, Monji A, et al. Oscillatory brain network changes after transcranial magnetic stimulation treatment in patients with major depressive disorder. J Affect Disord Rep. 2022;7:100277.

[24] Eldaief MC, Halko MA, Buckner RL, Pascual-Leone A. Transcranial magnetic stimulation modulates the brain’s intrinsic activity in a frequency-dependent manner. Proc Natl Acad Sci USA. 2011;108:21229–34. 22160708 10.1073/pnas.1113103109 PMC3248528

[25] Thut G, Miniussi C. New insights into rhythmic brain activity from TMS-EEG studies. Trends Cogn Sci. 2009;13:182–9. 19286414 10.1016/j.tics.2009.01.004

[26] Leuchter AF, Cook IA, Jin Y, Phillips B. The relationship between brain oscillatory activity and therapeutic effectiveness of transcranial magnetic stimulation in the treatment of major depressive disorder. Front Hum Neurosci. 2013;7:37. 23550274 10.3389/fnhum.2013.00037 PMC3581824

[27] Li CT, Chang WC, Cheng CM, Su TP, Bai YM, Tu PC. Comparative effects of 10-Hz rTMS and iTBS on cortico-striatal connectivity in major depressive disorder: a sham-controlled study. Mol Psychiatry. 2025;30:5084–92. 40581658 10.1038/s41380-025-03091-0

[28] Sheehan D, Lecrubier Y, Sheehan KH, Amorim P, Janavs J, Weiller E, et al. The Mini-International Neuropsychiatric Interview (M.I.N.I.): the development and validation of a structured diagnostic psychiatric interview for DSM-IV and ICD-10. J Clin Psychiatry. 1998;59:22–57. 9881538

[29] Beam W, Borckardt JJ, Reeves ST, George MS. An efficient and accurate new method for locating the F3 position for prefrontal TMS applications. Brain Stimul. 2009;2:50–4. 20539835 10.1016/j.brs.2008.09.006 PMC2882797

[30] Cao X, Deng C, Su X, Guo Y. Response and remission rates following high-frequency vs. Low-frequency repetitive transcranial magnetic stimulation (rTMS) over right DLPFC for treating major depressive disorder (MDD): A meta-analysis of randomized, double-blind trials. Front Psychiatry. 2018;9:413. 10.3389/fpsyt.2018.00413 PMC6137236 30245641

[31] Nolan H, Whelan R, Reilly RB. FASTER: Fully Automated Statistical Thresholding for EEG artifact Rejection. J Neurosci Methods. 2010;192:152–62. 20654646 10.1016/j.jneumeth.2010.07.015

[32] Nunez PL. Srinivasan Ramesh. Electric fields of the brain: the neurophysics of EEG. Oxford University Press; 2006.

[33] Schaefer A, Kong R, Gordon EM, Laumann TO, Zuo X-N, Holmes AJ, et al. Local-Global Parcellation of the Human Cerebral Cortex from Intrinsic Functional Connectivity MRI. Cereb Cortex. 2018;28:3095–114. 28981612 10.1093/cercor/bhx179 PMC6095216

[34] Gunnarsdottir KM, Li A, Smith RJ, Kang JY, Korzeniewska A, Crone NE, et al. Source-sink connectivity: A novel interictal EEG marker for seizure localization. Brain. 2022;145:3901–15. 36412516 10.1093/brain/awac300 PMC10200292

[35] Benjaminit Y, Hochberg Y. Controlling the false discovery rate: a practical and powerful approach to multiple testing. J. R. Stat. Soc. B. 1995;57:289–300.

[36] Rolls ET. The cingulate cortex and limbic systems for emotion, action, and memory. Brain Struct Funct. 2019;224:3001–18. 31451898 10.1007/s00429-019-01945-2 PMC6875144

[37] Tsai YC, Li CT, Liang WK, Muggleton NG, Tsai CC, Huang NE, et al. Critical role of rhythms in prefrontal transcranial magnetic stimulation for depression: A randomized sham-controlled study. Hum Brain Mapp. 2022;43:1535–47. 34873781 10.1002/hbm.25740 PMC8886663

[38] George MS, Huffman S, Doose J, Sun X, Dancy M, Faller J, et al. EEG synchronized left prefrontal transcranial magnetic stimulation (TMS) for treatment resistant depression is feasible and produces an entrainment dependent clinical response: A randomized controlled double blind clinical trial. Brain Stimul. 2023;16:1753–63. 38043646 10.1016/j.brs.2023.11.010 PMC10872322

[39] Pantazatos SP, Mclntosh JR, Saber GT, Sun X, Doose J, Faller J, et al. The timing of transcranial magnetic stimulation relative to the phase of prefrontal alpha EEG modulates downstream target engagement. Brain Stimul. 2023;16:830–9. 37187457 10.1016/j.brs.2023.05.007

[40] Tsai YC, Li CT, Juan CH. A review of critical brain oscillations in depression and the efficacy of transcranial magnetic stimulation treatment. Front Psychiatry. 2023;14:1073984. 10.3389/fpsyt.2023.1073984 PMC10228658 37260762

[41] Shibata S, Watanabe T, Yukawa Y, Minakuchi M, Shimomura R, Ichimura S, et al. Effects of transcranial static magnetic stimulation over the primary motor cortex on local and network spontaneous electroencephalogram oscillations. Sci Rep. 2021;11:8261. 10.1038/s41598-021-87746-2 PMC8050201 33859297

[42] Unnwongse K, Rampp S, Wehner T, Kowoll A, Parpaley Y, Von Lehe M, et al. Validating EEG source imaging using intracranial electrical stimulation. Brain Commun. 2023;5:fcad023. 10.1093/braincomms/fcad023 PMC9942548 36824389

[43] Fahimi Hnazaee M, Wittevrongel B, Khachatryan E, Libert A, Carrette E, Dauwe I, et al. Localization of deep brain activity with scalp and subdural EEG. Neuroimage. 2020;223:117344. 10.1016/j.neuroimage.2020.117344 32898677

[44] Liu S, Yang S, Wang C, Li J, Wang L. Effects of two types of repetitive transcranial magnetic stimulation on brain network in Parkinson’s disease. NPJ Parkinsons Dis. 2025;11:191. 40593930 10.1038/s41531-025-01054-4 PMC12218465

[45] Cash RFH, Weigand A, Zalesky A, Siddiqi SH, Downar J, Fitzgerald PB, et al. Using Brain Imaging to Improve Spatial Targeting of Transcranial Magnetic Stimulation for Depression. Biol Psychiatry. 2021;90:689–700. 32800379 10.1016/j.biopsych.2020.05.033

[46] Siddiqi SH, Taylor SF, Cooke D, Pascual-Leone A, George MS, Fox MD. Distinct symptom-specific treatment targets for circuit-based neuromodulation. Am J Psychiatry. 2020;177:435–46. 32160765 10.1176/appi.ajp.2019.19090915 PMC8396109

[47] Corlier J, Wilson A, Hunter AM, Vince-Cruz N, Krantz D, Levitt J, et al. Changes in Functional Connectivity Predict Outcome of Repetitive Transcranial Magnetic Stimulation Treatment of Major Depressive Disorder. Cereb Cortex. 2019;12:4958–67. 10.1093/cercor/bhz035 PMC7305800 30953441

[48] Bailey NW, Hoy KE, Rogasch NC, Thomson RH, McQueen S, Elliot D, et al. Responders to rTMS for depression show increased fronto-midline theta and theta connectivity compared to non-responders. Brain Stimul. 2018;11:190–203. 29128490 10.1016/j.brs.2017.10.015

