# Supplementary material for: Differential neural responses to rhythmic and patterned TMS protocols: Insights from EEG spectral analysis
Source: Neuropsychopharmacology. 2026 Jan 7;51(5):813–21. doi: 10.1038/s41386-025-02306-w (PMC13013849; doi:10.1038/s41386-025-02306-w)
Supplement: Supplementary file 1 — Supplementary Materials and Methods [file 41386_2025_2306_MOESM1_ESM.docx]

# Supplementary Materials and Methods

## Section 1: Subject Medication Details

11 of the 16 subjects were taking some medication within the three months prior to the start of treatment which could have affected neural activity. See Table S1 for exact subject counts. Due to small group counts, exact permutation tests were conducted to determine whether there were significant differences in the motor thresholds of those who were taking versus those who were not taking medications in each category. The tests did not reveal significant differences for anxiolytics (p = 0.279), antidepressants (p = 0.17), psycho-stimulants (p = 0.917), or hormones (p = 0.362). However, subjects taking mood stabilizers had significantly higher MTs than those who did not (p = 0.005).

| **Subject ID** | **Anxiolytics** | **Antidepressants** | **Mood Stabilizers** | **Psycho-Stimulants** | **Hormones** | **Total** |
| --- | --- | --- | --- | --- | --- | --- |
| **1** | Yes | Yes | Yes | Yes | Yes | 5 |
| **2** | No | Yes | No | No | Yes | 2 |
| **3** | No | No | No | No | No | 0 |
| **4** | No | Yes | Yes | No | Yes | 3 |
| **5** | No | No | No | No | No | 0 |
| **6** | No | Yes | No | Yes | No | 2 |
| **7** | Yes | Yes | Yes | Yes | No | 4 |
| **8** | No | Yes | No | Yes | No | 2 |
| **9** | No | Yes | No | No | Yes | 2 |
| **10** | Yes | Yes | No | Yes | No | 3 |
| **11** | No | No | No | No | No | 0 |
| **12** | No | Yes | Yes | No | No | 2 |
| **13** | No | No | No | Yes | No | 1 |
| **14** | No | No | No | No | No | 0 |
| **15** | No | No | Yes | No | No | 1 |
| **16** | No | No | No | No | No | 0 |
| **Total** | 3 | 9 | 5 | 6 | 4 | 27 |

Table S1 Subject medication use. "Yes" indicates that the patient was using a medication in the specified category within three months of their TMS treatment

## Section 2: EEG Preprocessing Details

A Piecewise Cubic Hermite Interpolating Polynomial (PCHIP) with a node spacing of 200 ms was used for detrending the recovery period after the last pulse of each train. PCHIP constructs a cubic Hermite interpolating polynomial on each subsegment of the data, ensuring that the first derivative of the interpolant agrees with an estimate of the derivative of the real data at each node. This derivative estimate is calculated in a way that enforces monotonicity of each spline, thus preventing spurious oscillations. EEG data were band pass filtered with a pass band of 1-55Hz, resampled to 1 kHz, and re-referenced to a common average. Data were then decomposed into independent components using independent component analysis (ICA). Noisy components were removed (rhythmic mean ± standard deviation: 4.7 components ± 1.4, patterned: 4.2 ± 1.5) and noisy channels (rhythmic: 3.7 channels ± 1.4, patterned: 4 ± 1.0) were then interpolated by their neighbors. M1 and M2 were pre-selected to always interpolated. Data was then visually inspected and epochs which still had visible non-physiological noise or artifacts were rejected (rhythmic: 2.4 epochs ± 2.6, patterned: 1.1 ± 1.5).

## Section 3: Power Calculation

To compute the power of the response frequency, the Gabor transform with a standard deviation of 3 cycles and 512 frequency segments were used, offering a balanced trade-off between time and frequency resolution. For each stimulation frequency, one-second time windows preceding and following the stimulation were used to generate the spectrogram, capturing the temporal evolution of the signal’s frequency content hence allowing to examine the differences induced by different stimulation frequencies.

## Section 4: Source Localization

## The inverse solution problem was calculated using compute the EEG signals generated by the brain sources. the Weighted minimum norm estimate (wMNE) with fixed dipole orientation and standardized low-resolution brain electromagnetic tomography (sLORETA) was used especially to compute deep structures contributions by adjusting the noise covariance matrix using a regularization parameter. The regularization parameter had a value of 1/SNR which was estimated to be around 15 dB in our preprocessed signals. The noise covariance matrix was computed from data that was recorded during a resting state baseline period while the patients were relaxed and before any TMS stimulations were administered. The regularization parameter was set to default value 0.2 to control the regularization process. To determine the position, orientation, and magnitude of dipolar sources, a fixed dipole orientation model was considered. The source dipoles were oriented orthogonally to the cortical surface, mimicking the orientation of pyramidal cells perpendicular to the cortex.

## Section 5: Connectivity

The process proceeds as presented in (1): for each 500 ms window, we constructed a discrete-time LTI model from the source EEG signals, which led to a sequence of models {$A_{j}$} for $j$ = 1, 2, ..., $M$, where $M$ is the total number of windows depending on the size of each epoch. Specifically, each recording from region $i$ was considered a realization of a state variable $x_{i}\left( t \right)$for $i$= 1, 2, ..., $N$ assumed to be generated by model $x(t+1) = A_{j}x(t)$ for $t \in[500(j-1), 500j].$ Each state evolution matrix $A_{j}$ was estimated by minimizing the squared error between the data and the model. That is, the cost function, $\left| \left| x\left( t \right)- \hat{x}\left( t \right) \right| \right|^{2}$ was minimized over $A_{j}$ such that $\hat{x}(t + 1) = A\hat{x}(t)$. The following variables describe a window of data:

• $T$ = window size (500 ms)

• $N$ = number of regions (100 in the Schaefer atlas)

• $b \in\mathbb{R}^{(T -1)N}$ , where $b$ are the region recordings at the next time point

• $H$ ∈ $\mathbb{R}^{(T -1)N\times N^{2}}$

• $X \in\mathbb{R}^{N\times N}$

where X is the vectorized adjacency matrix we are interested in $x_{i}(t)$, $t = 1, 2, ..., T,$ are the sources time series from each window of region $i$. The adjacency matrix model is formulated by expressing the data and unknowns as a linear system: $b = HX$. The unknown vector X is then estimated in MATLAB using the least squares solution: $X = H\backslash b$. The following linear system is derived by expanding the LTI model recursion across time steps:

$x_{1}(2) = A_{1,1}x_{1}(1) + A_{1,2}x_{2}(1) + ... + A_{1,N}x_{N}(1)$

$x_{2}(2) = A_{2,1}x_{1}(1) + A_{2,2}x_{2}(1) + ... + A_{2,N}x_{N}(1)$…

$$x_{N}(2) = A_{N,1}x_{1}(1) + A_{N,2}x_{2}(1) + ... + A_{N,N}x_{N}(1)$$

$x_{1}(3) = A_{1,1}x_{1}(2) + A_{1,2}x_{2}(2) + ... + A_{1,N}x_{N}(2)$

$x_{2}(3) = A_{2,1}x_{1}(2) + A_{2,2}x_{2}(2) + ... + A_{2,N}x_{N}(2)$ . . .

$x_{1}(T) = A_{1,1}x_{1}(T) + A_{1,2}x_{2}(T) + ... + A_{1,N}x_{N}(T)$. . .

$x_{N}(T) = A_{N,1}x_{1}(T) + A_{N,2}x_{2}(T) + ... + A_{N,N}x_{N}(T)$

The model matrices are constructed as such and solved for each window. $b= \left[ x_{1}\left( 2 \right), x_{2}\left( 2 \right)\ldots x_{N}\left( 2 \right)\ldots x_{1}\left( T \right), x_{2}\left( T \right)\ldots x_{N}(T) \right]^{T}$,


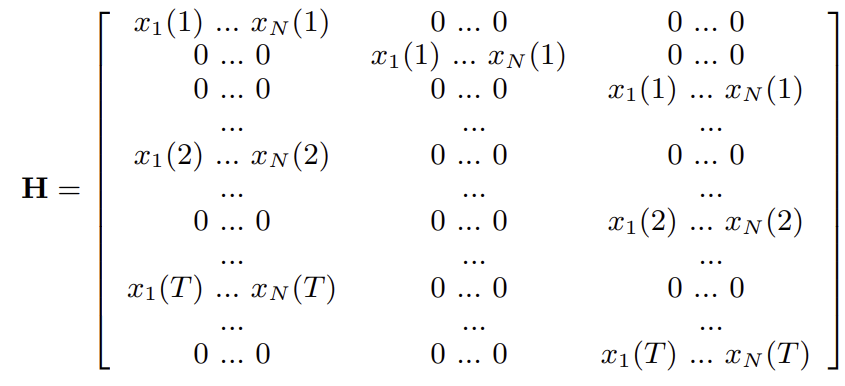


$X={[A_{1,1} A_{1,2}\ldots A_{1,N} A_{2,1}\ldots A_{N,N-1} A_{N,N} ]}^{T}$ After solving for $X$, it can be reshaped into a matrix A.

Supplemental Table Legends

**Table S2. Scalp-level percentage change in spectral power.** Cell values represent the percentage change in power from one second pre- to one second post-stimulation. Each column indicates the stimulation protocol and frequency administered and the response band observed, with the first letter of the column name denoting either Patterned or Rhythmic stimulation, the first Greek letter denoting the stimulation frequency band, and the second Greek letter denoting the response frequency band. For example, the column "R: α -> θ" lists alpha-rhythmic stimulation induced changes on the theta response band. *: p < 0.05, **: p < 0.01, ***: p < 0.001. (†): p-value significant after FDR correction across all tests conducted in the column.

**Table S3. Source-level percentage change in spectral power.** Cell values represent the percentage change in power from one second pre- to one second post-stimulation. Each column indicates the stimulation protocol and frequency administered and the response band observed, with the first letter of the column name denoting either Patterned or Rhythmic stimulation, the first Greek letter denoting the stimulation frequency band, and the second Greek letter denoting the response frequency band. For example, the column "R: α -> θ" lists alpha-rhythmic stimulation induced changes on the theta response band. *: p < 0.05, **: p < 0.01, ***: p < 0.001. (†): p-value significant after FDR correction across all tests conducted in the column.

**Table S4. Source-level percentage change in spectral power.** Cell values represent the percentage change in effective connectivity to the DLPFC from one second pre- to one second post-stimulation. Each column indicates the stimulation protocol and frequency administered and the response band observed, with the first letter of the column name denoting either Patterned or Rhythmic stimulation, the first Greek letter denoting the stimulation frequency band, and the second Greek letter denoting the response frequency band. For example, the column "R: α -> θ" lists alpha-rhythmic stimulation induced changes on the theta response band. *: p < 0.05, **: p < 0.01, ***: p < 0.001. (†): p-value significant after FDR correction across all tests conducted in the column.

**References**

1. Li A, Gunnarsdottir KM, Inati S, Zaghloul K, Gale J, Bulacio J, *et al.* (2017): Linear time-varying model characterizes invasive EEG signals generated from complex epileptic networks. *Proceedings of the Annual International Conference of the IEEE Engineering in Medicine and Biology Society, EMBS* 2802–2805.
